# Pharmaco-Multiomics: A New Frontier in Precision Psychiatry

**DOI:** 10.3390/ijms26031082

**Published:** 2025-01-26

**Authors:** Dhoha Dhieb, Kholoud Bastaki

**Affiliations:** Pharmaceutical Sciences Department, College of Pharmacy, QU Health, Qatar University, Doha P.O. Box 2713, Qatar; dhoha.dhieb@qu.edu.qa

**Keywords:** psychiatric disorders, pharmaco-multiomics, personalized psychiatry

## Abstract

The landscape of psychiatric care is poised for transformation through the integration of pharmaco-multiomics, encompassing genomics, proteomics, metabolomics, transcriptomics, epigenomics, and microbiomics. This review discusses how these approaches can revolutionize personalized treatment strategies in psychiatry by providing a nuanced understanding of the molecular bases of psychiatric disorders and individual pharmacotherapy responses. With nearly one billion affected individuals globally, the shortcomings of traditional treatments, characterized by inconsistent efficacy and frequent adverse effects, are increasingly evident. Advanced computational technologies such as artificial intelligence (AI) and machine learning (ML) play crucial roles in processing and integrating complex omics data, enhancing predictive accuracy, and creating tailored therapeutic strategies. To effectively harness the potential of pharmaco-multiomics approaches in psychiatry, it is crucial to address challenges such as high costs, technological demands, and disparate healthcare systems. Additionally, navigating stringent ethical considerations, including data security, potential discrimination, and ensuring equitable access, is essential for the full realization of this approach. This process requires ongoing validation and comprehensive integration efforts. By analyzing recent advances and elucidating how different omic dimensions contribute to therapeutic customization, this review aims to highlight the promising role of pharmaco-multiomics in enhancing patient outcomes and shifting psychiatric treatments from a one-size-fits-all approach towards a more precise and patient-centered model of care.

## 1. Introduction

Psychiatric disorders encompass a diverse range of conditions that profoundly impact cognitive, emotional, perceptual, behavioral, and mood-related functioning. This broad category includes depressive disorders, bipolar disorder, anxiety disorders, trauma- and stressor-related disorders, psychotic disorders, dissociative disorders, eating disorders, and personality disorders, alongside neurodevelopmental disorders such as ADHD (attention-deficit/hyperactivity disorder) and autism spectrum disorders [1,2,3,4]. These illnesses are often characterized by significant distress, social dysfunction, and a decline in quality of life, underscoring the fundamental importance of mental health for personal well-being, interpersonal relationships, and societal contributions [5]. The ramifications of mental health issues extend beyond psychological suffering, often exacerbating physical ailments and increasing both disability and mortality rates [6,7]. In 2019, 970 million people worldwide, or one in every eight individuals, were affected by mental disorders, with anxiety and depressive disorders identified as the most prevalent [8]. The COVID-19 pandemic further exacerbated these issues, leading to a substantial increase in anxiety and depressive disorders by 26% and 28%, respectively, in 2020 [9]. Regionally, the situation in Qatar mirrors global trends, with about 20% of adults attending primary care facilities presenting diagnosable mental disorders, predominantly anxiety and depression [10,11]. Notably, Qatar ranked second in the Middle East for the increased burden of premature mortality from mental health disorders, highlighting a significant regional health challenge [10]. Economically, the impact of mental disorders is significant. Financial losses due to mental health issues were estimated at about USD 1.3 trillion in 2010, rising to USD 1.6 trillion in 2019, with projections indicating an increase to nearly USD 2.5 trillion by 2030 [12]. Despite their high prevalence and cost, the effectiveness of pharmacological treatments like antidepressants remain limited. In clinical trials, these medications showed modest response rates of only 42% to 53% for major depressive disorder, affecting an estimated 163 million individuals [13,14]. Furthermore, a substantial proportion of patients, up to 60%, display poor responses and achieve low remission rates after initial drug administration, accompanied frequently by severe adverse drug reactions (ADRs) [15,16,17,18]. These ADRs themselves are responsible for a significant number of hospital admissions, representing between 5% and 10% of cases, thereby elevating both the clinical and economic burdens of mental health care [19] and significantly increasing healthcare costs. In the United States, the annual cost associated with these reactions can reach up to USD 30.1 billion [20]. This underscores the shortcomings of the prevailing trial-and-error approach in psychiatric medication management, an approach closely associated with poor treatment adherence and discontinuation [20,21]. Understanding the variability in patient responses is crucial, as it stems from a complex interplay of genetic, environmental, pathophysiological, and dietary factors. Specifically, genetic differences play a significant role in influencing drug efficacy and the likelihood of ADRs [22,23]. Research indicates that 15% to 30% of the variability in treatment response can be attributed to genetic alleles affecting drug absorption, metabolism, transport, and mechanism of action [20]. Consequently, identifying these specific genetic variants can predict drug efficacy and potential adverse reactions, thereby enhancing treatment outcomes [24]. This evidence collectively mandates a paradigm shift towards personalized medicine in psychiatry. Embarking on this transformative path necessitates embracing a comprehensive approach incorporating the diverse biological and molecular factors that shape individual patient experiences [25,26,27]. Through integration of multiple omics layers, clinicians and researchers can unearth previously inaccessible insights, substantially enriching our molecular understanding of psychiatric disorders [28]. Utilizing extensive datasets from extensive pharmaco-multiomics investigations propels psychiatry towards more precise, personalized therapeutic interventions. These enhance clinical outcomes and provide patient-specific care tailored to improving clinical outcomes and aligning treatments with individual biological and environmental contexts. In this review, we discuss recent findings across various omics layers and underscore their implications for the development of personalized treatment strategies in psychiatry. We detail how each omic contributes to the creation of targeted therapeutic approaches, emphasizing its role in promoting personalized care and improving patient outcomes. Ultimately, our examination aims to highlight the transformative potential of pharmaco-multiomics in establishing sophisticated, patient-centered therapeutic strategies that not only accommodate the biological individuality of each patient but also set new paradigms in the management of psychiatric disorders.

## 2. Pharmacogenomics in Psychiatry

Pharmacogenomics (PGx) stands at the frontier of personalized medicine in psychiatry, dedicated to understanding how genetic variation affects individual responses to psychiatric drugs [29]. Over the past four decades, extensive research focusing on both direct and indirect evidence has underscored the clinical utility of PGx in guiding medication dosing and selection decisions, particularly for mental health treatments [30]. This research has examined a multitude of genetic variants associated with drug targets such as the serotonin reuptake transporter (SLC6A4), serotonin receptors (HTR2A and HTR2C), and the dopamine D2 receptor (DRD2). Additional significant pharmacogenes include transporter genes like ATP-binding cassette subfamily B member 1 (ABCB1) and organic cation transporter 1 (OCT1). The study of drug-metabolizing enzymes such as cytochrome P450 2C19 (CYP2C19), cytochrome P450 2D6 (CYP2D6), cytochrome P450 2B6 (CYP2B6), and catechol-O-methyltransferase (COMT) has also been extensive. Moreover, the major histocompatibility complex genes, including HLA-A and HLA-B, have been recognized for their roles related to immunogenic responses potentially triggered by psychiatric medications. This broad array of genes and their variants has been incorporated into PGx-driven evidence-based guidelines for many psychiatric drugs, including antidepressants [31,32,33], antipsychotics [34], ADHD treatments [35,36], and mood stabilizers [37]. The integration of genetic testing into prescribing practices has become increasingly necessary, paving the way for personalized medicine. The International Society of Psychiatric Genetics (ISPG) highlights the importance of pharmacogenetic testing in psychiatry, specifically recommending genetic testing for enzymes such as CYP2C19 and CYP2D6. This testing is particularly valuable for patients who have previously exhibited inadequate responses or adverse reactions to certain antidepressants and antipsychotics [38]. The U.S. Food and Drug Administration (FDA) and the Clinical Pharmacogenetics Implementation Consortium (CPIC) advocate for genetic testing prior to the prescription of specific drugs like carbamazepine to optimize therapeutic outcomes and mitigate adverse effects [39]. Guidelines supported by the CPIC along with international resources such as the Dutch Pharmacogenetics Working Group (DPWG) and the Pharmacogenomics Knowledgebase (PharmGKB) are instrumental in translating PGx findings into clinical practice [40,41]. The FDA has also developed a reference table of pharmacogenetic associations, an essential tool for healthcare providers to evaluate drug–gene interactions for optimal therapeutic strategies, including medication dosage and avoidance of toxicities. These guidelines and resources have significantly influenced the labeling of many psychiatric medications in relation to specific genotypes of CYP2C19 and CYP2D6 [40,42]. Despite these advancements, consistent implementation of these guidelines across different clinical settings remains a critical challenge [43]. Pharmacogenomics has uncovered substantial variations in drug pharmacokinetics and pharmacodynamics across individuals and ethnic groups. The acknowledgment of these differences is crucial for optimizing drug dosages, minimizing ADRs, and enhancing overall treatment outcomes. This section of our review pertains to pharmacogenomics, and particularly focuses on the influence of the CYP450 gene family and the HLA gene family in psychiatry, given their proven clinical relevance. By leveraging the actionable insights provided by genotyping these genes, we can refine and personalize treatment strategies, potentially revolutionizing psychiatric care by aligning drug therapies more closely with individual genetic profiles.

### 2.1. Cytochrome P450 Variability and Its Implications in Psychiatric Pharmacotherapy

Genetic polymorphisms within CYP450 enzymes introduce significant variability in drug metabolism, thereby impacting therapeutic efficacy and the risk of adverse drug reactions (ADRs) [44,45]. These variations create distinct metabolic phenotypes among patients, ranging from poor to ultra-rapid metabolizers, each exhibiting unique responses to medication [46]. Poor metabolizers (PMs), for example, may experience more adverse reactions at standard doses, due to reduced enzyme activity from homozygous non-functional alleles or gene deletion [47]. Conversely, ultra-rapid metabolizers (UMs) might show an inadequate response to drugs administrated at standard doses because of elevated enzyme activity due to multiple active gene copies, posing significant challenges in attaining therapeutic efficacy [48]. Moreover, genetic mutations such as alternative splicing or frame-shifting events can profoundly alter enzyme structure and function. These alterations consequently affect the metabolism of key psychiatric drugs, adding another layer of complexity to managing patient-specific treatment plans [49]. Table 1 presents a comprehensive synthesis of psychotropic medications, including actionable and informative genes, alongside recommendations from the Clinical Pharmacogenetics Implementation Consortium (CPIC) and the Dutch Pharmacogenetics Working Group (DPWG). All data are sourced from the Pharmacogenomics Knowledgebase (PharmGKB). This table is instrumental in guiding clinicians on genotype-specific drug recommendations, enhancing the precision of psychiatric pharmacotherapy. This underlines the critical need to incorporate pharmacogenetic testing into routine psychiatric practice. By tailoring drug dosages and therapy plans to individual genetic profiles, healthcare providers can optimize treatment outcomes and minimize adverse effects, advancing towards a more personalized approach in psychiatry [25,32].

#### 2.1.1. CYP450 Genetic Variations and Antidepressant Treatment

Antidepressants, particularly selective serotonin reuptake inhibitors (SSRIs) and serotonin–norepinephrine reuptake inhibitors (SNRIs), are metabolized extensively by the CYP450 enzyme system. These pharmacotherapeutic agents serve as frontline treatments for a variety of anxiety and depressive disorders [50,51,52]. SSRIs, which include fluoxetine, citalopram, escitalopram, paroxetine, sertraline, and fluvoxamine, share a mechanistic pathway that involves the inhibition of serotonin reuptake, resulting in increased synaptic serotonin levels [53]. Notably, each SSRI is characterized by distinct pharmacokinetic profiles, pharmacodynamics attributes, and a unique spectrum of side effects, emphasizing the need for individualized therapeutic considerations [53].

The role of specific CYP450 enzymes in the metabolism of SSRIs has been extensively documented. Cacabelos et al. noted that approximately 24% of these antidepressants are metabolized by CYP1A2, 5% by CYP2B6, 38% by CYP2C19, 85% by CYP2D6, and 38% by CYP3A4 [54]. All SSRIs inhibit CYP2D6, with fluoxetine and paroxetine being the most potent inhibitors, though interactions with CYP3A4 are generally insignificant [55]. Citalopram and escitalopram are primarily metabolized by the highly polymorphic enzyme CYP2C19. The Clinical Pharmacogenetics Implementation Consortium (CPIC) has established gene-based therapeutic guidelines for these SSRIs based on the *CYP2C19* genotype [56,57]. Genetic variations in CYP2C19 can significantly alter the enzyme’s metabolic activity, affecting drug plasma concentrations. For instance, the CYP2C19*1 allele encodes a fully functional enzyme, while the *17 variant is associated with higher activity and the *2 variant with no activity. Patients with poor metabolizing phenotypes exhibit high plasma concentrations of citalopram, prompting FDA recommendations for a 50% dose reduction to avoid the risk of QT prolongation [58,59,60]. Similarly, fluvoxamine is metabolized by CYP2D6. CPIC guidelines recommend a 25–50% initial dose reduction for fluvoxamine in poor metabolizers, allowing for titration to the maximum effective dose while minimizing adverse effects, or considering alternative medications not metabolized by CYP2D6 [58,61]. Similarly, SNRIs present complex metabolic profiles requiring careful consideration of drug–drug interactions and patient-specific factors [62,63]. For example, venlafaxine and duloxetine are extensively metabolized by CYP2D6, with variations in this enzyme drastically affecting their pharmacokinetic behaviors and necessitating individualized dosing schedules, especially in patients with hepatic or renal impairments [64,65,66]. The pharmacokinetics of newer agents such as levomilnacipran, which is preferentially metabolized by CYP3A4, also exemplifies the intricate considerations required in managing treatments due to its significant impact on norepinephrine compared to serotonin reuptake [58,61]. This detailed attention to metabolic pathways ensures efficacy and safety, particularly in populations with genetic predispositions affecting enzyme activity. Additionally, Tricyclic antidepressants (TCAs), such as imipramine, amitriptyline, trimipramine, and clomipramine, represent some of the earliest forms of antidepressant drugs and are primarily metabolized by the CYP2C19 enzyme. However, their biotransformation into active or inert metabolites crucially depends on subsequent processing by the CYP2D6 enzyme [67,68]. This dual-stage metabolism not only illustrates the complexity of drug interactions within the body but also highlights how genetic variations influencing these enzymes can significantly affect both the efficacy and safety profiles of these medications. In contrast to TCAs’ reliance mainly on two cytochrome P450 enzymes for their metabolism, newer serotonin modulators like Mirtazapine involve a broader spectrum of these isoenzymes in its metabolic pathway, including CYP2D6, CYP1A2, and CYP3A4 [69]. In the context of treatment-resistant depression, ketamine and esketamine introduce complex considerations due to genetic polymorphisms affecting their metabolism. These agents are primarily metabolized by polymorphic enzymes such as CYP2B6, CYP3A4, CYP2A6, CYP2C9, and CYP3A5, which influence the conversion of ketamine to norketamine (NK) and then to hydroxynorketamine (HNK), as well as the transformation of esketamine to noresketamine and hydroxynoresketamine. Notably, CYP2B6, which exhibits numerous single-nucleotide polymorphisms leading to 38 variant proteins, demonstrates varying catalytic activities that significantly impact drug exposure and pharmacokinetics. Specifically, decreased-function CYP2B6 variants like CYP2B6*6 and CYP2B6*9 show reduced metabolic activity, directly affecting the efficacy and safety of treatments. Studies indicate that the wild-type CYP2B6 enzymes exhibit the highest activity for the N-demethylation of ketamine and its derivatives, with some variant enzymes showing up to 35% lower activity compared to the wild type. Such genetic discrepancies highlight the necessity for personalized therapeutic strategies similar to those developed for SSRIs and SNRIs based on CYP2C19 and CYP2D6 variations [70]. These insights into the pharmacogenomics of ketamine and esketamine underscore the critical need for clinical validation of genetic influences on therapy outcomes. Understanding these genetic and pharmacological profiles is essential to optimizing treatment strategies in personalized psychiatry, ensuring efficacy and safety for individuals with specific genetic predispositions affecting metabolic enzyme activity.

#### 2.1.2. CYP450 Genetic Variations and Antipsychotic Treatment

Antipsychotics, which are pivotal for managing psychiatric conditions such as schizophrenia, bipolar disorder, and major depression, demonstrate significant pharmacogenetic diversity. Approximately 40% of these drugs are metabolized by the polymorphic enzyme CYP2D6, whose variants, along with those in CYP2C19, critically influence drug metabolism, efficacy, and safety. Key enzymes involved in antipsychotic metabolism include CYP1A2, CYP2D6, CYP3A4, and CYP3A5, each contributing uniquely to these medications’ metabolic pathways [71,72,73]. CYP2D6 plays a primary role in the metabolism of antipsychotics like aripiprazole and risperidone, where genetic polymorphisms can result in a range of metabolic phenotypes from poor to ultra-rapid metabolizers, significantly affecting drug exposure and therapeutic efficacy [74,75]. Moreover, CYP1A2 is essential for the metabolism of medications such as olanzapine, clozapine, and asenapine, with genetic variants potentially altering plasma concentrations and increasing the risk of side effects [76,77]. The enzyme CYP3A4, responsible for the metabolism of several antipsychotics, harbors mutations like the rs680055 variant, which can profoundly impact drug response [77,78]. Less prevalent but still influential, *CYP3A5* variations affect drug ratios and plasma levels essential for optimal therapeutic outcomes [79,80]. Additionally, the *ABCB1* gene encodes transporters crucial for drug efflux, affecting drug exposure, with variants such as rs1045642 and rs1128503 modifying the clearance and plasma levels of antipsychotics [80,81,82,83]. The interplay of these enzymes underscores the complexity of antipsychotic drug metabolism, highlighting the importance of personalized treatment approaches. For example, poor metabolizers of CYP2D6 may experience higher plasma drug concentrations, necessitating dosage adjustments to mitigate side effects. The FDA has issued dosing recommendations for 24 antipsychotic drugs based on the CYP2D6 phenotype, illustrating the clinical relevance of genetic profiling [74,75]. While pharmacogenetic testing offers significant potential for enhancing treatment outcomes, its role in predicting adverse reactions remains uncertain. Research in this domain has yielded mixed outcomes, underscoring the complexity of genetic influences on drug response. For example, studies show that CYP450 gene genotyping has limited utility in predicting adverse reactions for drugs like nortriptyline or escitalopram [84]. Conversely, other evidence suggests improved outcomes with pharmacogenetically guided treatment decisions, as one study reported a 23% improvement when treatment was tailored based on genetic testing, with no cases of worsened conditions [73]. Nonetheless, some studies indicate that pharmacogenetic analysis may not significantly reduce certain side effects, such as hyperprolactinemia, which highlights the need for further research in this area [85]. Aside from CYP450 enzymes, genetic variations in other molecular targets such as the *HTR2C* gene and the DRD2*A1 allele also modulate the efficacy and side effect profiles of antipsychotics. For example, variations in HTR2C are associated with metabolic disturbances and weight gain, whereas the DRD2*A1 allele can influence prolactin levels in patients treated with antipsychotics such as clozapine [86,87].

#### 2.1.3. CYP450 Genetic Variations and Mood Stabilizer Treatments

The treatment efficacy and safety of mood stabilizers, pivotal in managing bipolar disorder, are significantly influenced by genetic variations [88]. Drugs such as valproic acid (VPA), carbamazepine (CBZ), and lithium each have distinct metabolic pathways affected by genetic predispositions. Valproic acid is primarily metabolized by CYP2C9, along with contributions of 20–25% from CYP2B6 and CYP2A6 enzymes, but notable variants like CYP2C19*3 and CYP2C19*2 are particularly impactful [89]. These variants alter plasma concentrations of VPA and are linked to an increased risk of side effects, such as significant weight gain observed in Japanese women. Additionally, the CYP2C9*3 variant is associated with the formation of hepatotoxic metabolites, presenting further challenges in the clinical management of VPA dosing. Carbamazepine metabolism involves a more extensive network of enzymes, including CYP2C8, CYP3A4, and epoxide hydrolase gene (*EPHX1*), which is crucial for converting CBZ into its active metabolite. Variants such as the non-functional CYP3A5*3 allele significantly affect CBZ plasma levels and, by extension, patient responses to therapy, illustrating the critical need for genetic profiling in optimizing drug dosages and reducing adverse effects [90,91,92,93]. Lithium, uniquely among mood stabilizers, does not undergo metabolism by the CYP450 system, as it is excreted renally. Nevertheless, the long-term use of lithium is associated with potential impairments in thyroid and renal function, highlighting the importance of monitoring and managing these effects during treatment [90,91,92].

#### 2.1.4. Human Leukocyte Antigen (HLA) Gene and Psychiatric Treatment

The human leukocyte antigen (HLA) gene family encodes major histocompatibility complex (MHC) proteins, which are crucial for immune and inflammatory responses [94]. Recent studies highlight the significance of HLA genetic variations in the etiology of psychiatric disorders such as bipolar disorder and schizophrenia, though not in depressive disorders and ADHD [95,96]. HLA molecules modulate neural signaling and synaptic integration, influencing behaviors, learning, and memory [97]. Specific HLA alleles have been linked to psychotropic treatment responses and the development of ADRs. For instance, certain Class I and II HLA alleles are implicated in clozapine-induced agranulocytosis [98] and correlate with better treatment outcomes for risperidone in schizophrenia patients due to specific amino acid variants in the HLA-A peptide-binding groove [99]. Additionally, HLA polymorphisms are associated with severe cutaneous adverse reactions (SCARs), like Stevens–Johnson syndrome (SJS) and toxic epidermal necrolysis (TEN), particularly in Asian populations treated with anticonvulsants such as carbamazepine and oxcarbazepine [100], and phenytoin, which are linked to biomarkers HLA-B*15:02 and HLA-A*31:01 [101]. These severe reactions include blistering rashes, hemorrhagic erosions of mucous membranes, skin detachment, fever, and internal organ lesions [102]. The nucleotide sequences of HLA-A and HLA-B are highly polymorphic, with hundreds of variants that influence susceptibility to various diseases and adverse reactions to pharmaceuticals [101,103]. Given the significant impact of HLA genetic diversity on treatment outcomes and ADRs in psychiatry, incorporating HLA genotyping into pharmacogenomic panels for psychiatric patients can enhance individualized therapy. Preemptive HLA testing could help prevent life-threatening side effects and optimize treatment efficacy, thereby improving overall patient care.

## 3. Pharmacometabolomics in Psychiatry

While pharmacogenomics has been instrumental in advancing precision medicine by predicting drug responses based on genetic profiles, it does not fully capture the influence of environmental and lifestyle factors such as diet, age, nutrition, gender, and the gut microbiome [104], which substantially impact drug efficacy and safety. Introduced in 2006, pharmacometabolomics fill this gap by analyzing both baseline and dynamic post-treatment metabolic profiles, thereby enhancing the predictability of individual drug response variations [105].

A previous study encompassed nearly 800 patients diagnosed with major depressive disorder (MDD) who were treated with the selective serotonin reuptake inhibitors (SSRIs) citalopram and escitalopram. Clinical depression symptoms were evaluated using the Hamilton Depression Rating Scale (HAMD) and the Quick Inventory of Depressive Symptomatology–Clinician Rated (QIDS-C16) at baseline and after 4 and 8 weeks of treatment. Genomic and metabolomic analyses were conducted on blood samples collected at these time points. Pharmacometabolomics-informed pharmacogenomic analyses in this context have explored both exogenous and endogenous metabolomics. A genome-wide association study (GWAS) of 529 patients has been conducted to uncover genetic variants associated with drug response phenotypes such as remission and response, considering covariates such as age, sex, drug, and dose [106,107]. One significant study identified SNPs related to blood concentrations of escitalopram and its metabolite S-didesmethylcitalopram. This GWAS identified significant correlations between SNPs in the CYP2C19 and CYP2D6 genes with the concentrations of escitalopram and S-didesmethylcitalopram, respectively, both of which are enzymes known to metabolize these drugs. Interestingly, the study also pinpointed a novel association with the TRIML1 gene, suggesting its potential involvement in drug metabolism [107,108]. In addition, the PGRN-AMPS study also investigated endogenous metabolites. One pivotal metabolomic analysis focused on glycine within the nitrogen metabolism pathway. In this analysis, plasma samples from 40 patients (20 escitalopram remitters and 20 non-remitters) were screened using a semi-quantitative gas chromatography–mass spectrometry platform. Glycine was identified as significantly associated with clinical response, prompting a follow-up with a tag SNP genotyping study across glycine metabolism pathway genes in a larger cohort of 512 MDD patients. This study revealed associations between SNPs in the GLDC gene and several clinical outcomes, a finding further validated in a replication cohort and functional genomic studies demonstrating altered protein binding in central nervous system-derived cell lines [106]. Subsequent research targeted serotonin, a key metabolite involved in the mechanism of action of SSRIs. Using plasma samples from 290 patients, a quantitative, targeted liquid chromatography–electrochemical coulometric array (LCECA) platform was employed to analyze metabolites primarily from the tryptophan, tyrosine, and tocopherol pathways. Among the 31 metabolites identified, serotonin levels were most significantly correlated with SSRI clinical outcomes. Consequently, baseline serotonin concentrations and their changes after 4 or 8 weeks of SSRI treatment were used as phenotypes for GWAS. This serotonin-focused GWAS identified two major SNP signals in the TSPAN5 and ERICH3 genes, both of which were associated with plasma serotonin levels. Functional genomic studies further revealed that these SNPs influenced serotonin concentrations in cell culture media, highlighting their potential functional relevance in SSRI response [109,110,111].

On the other hand, the data derived from metabolomic studies are pivotal in bridging critical gaps, as they map the regulation of metabolic pathways. This mapping is essential for illustrating interactions between genome-encoded proteins and environmental factors such as medication exposure. As such, the metabolome acts as a dynamic indicator of an individual’s metabolic status at specific times or in response to certain environmental stimuli. Metabolomic studies on ketamine and esketamine have shown that these antidepressants alter key metabolic pathways, such as the tricarboxylic acid cycle and lipid metabolism, influencing mitochondrial function and neurotransmission. These findings, derived from both preclinical and clinical studies, suggest that metabolic changes are integral to the antidepressant effects observed with these compounds. As research advances, these insights could inform personalized treatment strategies for treatment-resistant depression, enhancing clinical management approaches [112]. A focus in untargeted metabolomic studies, particularly within the realm of antipsychotic treatments, reveals frequent associations with carboxylic acids, a group that includes essential molecules like amino acids, citric acid cycle intermediates, and fatty acids. These compounds are vital for neurotransmitter synthesis and functionality [113]. Research underscores the potential links between amino acids and both the pathophysiology of schizophrenia and the therapeutic responses to antipsychotic drugs [114]. Genetic studies linking amino acid binding efficiency with schizophrenia symptoms have spurred further research into the potential therapeutic applications of amino acids and related compounds [115]. Furthermore, studies have explored other metabolite classes like keto acids and organic compounds in the context of antipsychotic treatments. Keto acids, which feature ketone and carboxylic acid groups, are influenced by both medications and dietary factors [116,117]. In some cases, ketogenic diets have shown potential in managing antipsychotic-associated hyperglycemia and improving symptoms. Organic compounds, particularly those containing organooxygen like glucose and glucuronic acid, and organonitrogen compounds such as trimethylamine N-oxide (TMAO), have been linked to the efficacy and side effects of antipsychotics, including their effects on metabolic and cardiovascular health [118,119,120]. Targeted metabolomic studies have highlighted acylcarnitines as important elements associated with antipsychotic use, implicated in various biological functions, including energy production, lipid metabolism, inflammation, and insulin sensitivity [121,122,123,124,125]. Additionally, these studies have found significant changes in neurotransmitter levels in plasma, suggesting their potential as biomarkers for psychopharmacological treatments [126,127]. However, discrepancies observed in brain sample analyses point to the need for further research to clarify these findings [128].

Pharmacometabolomics adds a crucial dimension to the broader field of pharmacogenomics by offering a more intricate understanding of how metabolic profiles and genetic polymorphisms interact to influence drug efficacy and safety. The continual advancements in metabolomics analytical platforms and their integration with other “omics” data ensure that this field will remain at the forefront of personalized medicine, offering new opportunities to optimize therapeutic outcomes for complex disorders.

## 4. Pharmacotranscriptomics in Psychiatry

The quest for novel targets and reliable biomarkers to predict therapeutic efficacy in psychiatric treatments remains a significant challenge [129,130]. A deeper understanding of how antidepressants modulate gene expression over time in key tissues could dramatically refine treatment strategies and spearhead the development of more effective therapies [131]. RNA, as the immediate product of gene expression and epigenetic modifications, serves as an accurate indicator of an individual’s functional status, making it a prime target for research studies [132]. Studies have compared peripheral RNA expression profiles between patients with major depression and healthy controls, as well as across varied treatment stages. Such comparisons aim to identify potential biomarkers that can prognosticate treatment responses [133].

The complexity of human studies, influenced by variables such as age and comorbidities, requires large cohorts for consistent biomarker identification. Conversely, animal models offer a controlled environment to study antidepressant effects, allowing for precise manipulation of treatment variables [134,135]. These models facilitate deeper cellular and molecular investigations, enhancing our understanding of psychiatric disorders [135,136]. Recent efforts using animal models have focused on deciphering mechanisms behind stress vulnerability and resilience, particularly looking at transcriptional signatures [137,138,139,140,141]. These studies have identified a varied response to treatments, enabling the selection of animal subjects that showcase strong antidepressant effects for further studies on the biological pathways involved in behavioral normalization [142]. Despite the prevalent use of SSRIs in treating major depression, there is a notable scarcity of comprehensive reviews concerning their transcriptional effects in animal models [143,144]. This gap highlights an urgent need to identify common gene targets across species, which could enhance therapeutic effectiveness. Large-scale transcriptome studies have revealed numerous differentially expressed genes in conditions like schizophrenia, particularly in the dorsolateral prefrontal cortex [145,146]. Yet, interpreting these changes remains complicated due to confounders such as antipsychotic drug exposure [147,148].

Pharmacotranscriptomics seeks to elucidate the interactions between drug effects and psychiatric disorders by investigating the transcriptional impact of antidepressants and antipsychotic drugs. Interestingly, these drugs can induce gene expression changes that either reflect or counter the alterations observed in disorders like schizophrenia [149,150,151,152,153]. This complexity underscores the importance of a nuanced understanding of how these medications influence the brain’s transcriptome, informing both treatment strategies and drug development. Transcriptome-wide association studies (TWAS) provide a methodology to directly quantify the impact of gene expression on psychiatric traits. This approach simplifies complex genetic data into gene-level insights, having already offered significant revelations concerning conditions such as ADHD, major depression, treatment-resistant depression, and autism spectrum disorder [154]. Furthermore, transcriptomic data can streamline drug repurposing efforts by utilizing open-source databases that depict interactions between existing drugs and specific gene products. This strategy can accelerate the drug development process, reducing some of the costs and time typically required [155,156,157]. Given the high incidence of comorbidity in psychiatric conditions and frequent polypharmacy, identifying drugs that target consistent gene expression patterns across various disorders could optimize treatment approaches and minimize the need for multiple medications [154].

## 5. Pharmacomicrobiomics in Psychiatry

The exploration of pharmacomicrobiomics within the realm of personalized psychiatry offers a promising frontier in enhancing the efficacy and specificity of psychiatric treatments [158]. Pharmacomicrobiomics research has highlighted that gut microorganisms affect psychotropic drug absorption, distribution, metabolism, and excretion, leading to interindividual variability in therapeutic outcomes and adverse effects [159]. For instance, variations in microbial composition can influence the pharmacokinetics of antidepressants and antipsychotics, altering their availability and action [159,160].

The gut microbiome significantly influences psychiatric drug efficacy through mechanisms such as bioaccumulation and modulation of metabolic pathways. Strains like Streptococcus salivarius can uptake drugs like aripiprazole and duloxetine, affecting their availability and, hence, treatment efficacy, a process further complicated by interactions like cross-feeding, which can alter microbial composition [161]. Moreover, the gut microbiome impacts brain function indirectly by modifying tryptophan metabolism through the kynurenine pathway, a process linked to conditions like major depressive disorder and schizophrenia [161]. This interaction not only alters neurotransmitter levels such as serotonin but also influences the efficacy of SSRIs, with drugs like escitalopram increasing beneficial microbial metabolites in patients [162].

A broad spectrum of studies collectively illuminate the intricate role of the gut microbiome in modulating drug responses. Foundational research such as the Human Microbiome Project, an initiative by the NIH, provides a comprehensive catalog of microbial profiles, laying the groundwork for understanding microbiome variations that influence pharmacological outcomes [163]. Notably, European initiatives like ONCOBIOME and The Global Microbiome Conservancy extend this understanding by focusing on the microbiome’s role in specific health conditions, including psychiatric disorders [164]. Cussotto et al. (2019) underscores how psychotropic drugs such as antidepressants and antipsychotics can induce significant shifts in gut microbiota composition, which in turn can affect drug efficacy and tolerability [165]. In addition, there is increasing emphasis on the bidirectional interaction between drugs and the gut microbiome, highlighting that microbes can metabolize drugs, and conversely, drugs can modify the gut microbiota composition [166]. One study reviews the effects of atypical antipsychotics on the gut microbiome, suggesting that treatments themselves can influence microbial compositions, thus potentially opening up new avenues for therapeutic strategies [167]. Despite the promising insights these studies provide, the field acknowledges significant variability and challenges in methodology, as pointed out in systematic evaluations using tools like the STORMS checklist. Future research directions emphasize the need for more stringent clinical studies and the development of standardized protocols to reliably harness the gut microbiome’s potential in improving psychiatric treatment outcomes.

Thus, pharmacomicrobiomics stands at the forefront of a transformative shift in precision psychiatry, where thorough scientific exploration promises to unlock tailored therapeutic strategies that consider the microbiome a central player in drug response variability. The potential role of gut microbiota-derived metabolites as biomarkers, as highlighted in various studies, presents another promising avenue for research. Such metabolites might not only clarify the microbiome’s impact on drug responses but also offer new targets for therapeutic intervention, potentially leading to the development of novel psychobiotics [168]. Future research efforts will likely focus on elucidating the mechanisms of gut microbiota to brain communication, which emphasize gut neuropeptides and their systemic effects [169]. The intricate interplay between the gut microbiome and psychiatric pharmacology, supported by a robust foundation of research, heralds a future where treatments are not only centered on individual genetic makeup but also tailored to microbial profiles. This integrated approach promises more effective management of psychiatric disorders, with fewer side effects and improved patient outcomes, marking a significant advance in the pursuit of precision psychiatry. Harnessing the microbiome’s role in pharmacology could revolutionize treatment personalization, allowing clinicians to predict patient responses to drugs and potentially adjust therapies based on microbiome profiles. Despite promising findings, inconsistency in methodology and the need for robust study designs remain challenges. Thus, future research in pharmacomicrobiomics aims to establish clearer guidelines and integrate microbiome analysis into clinical practice, ultimately enhancing psychiatric care through personalized medicine.

## 6. Pharmacoepigenomics in Psychiatry

Pharmacoepigenomics in psychiatry elucidates the complex interplay between epigenetic mechanisms such as DNA methylation (DNAm) and histone modifications, thereby advancing our understanding of drug responses and therapeutic outcomes in mental health disorders [170]. Epigenetic alterations at gene promoters generally reduce gene expression, whereas methylation within gene bodies may enhance it. These modifications variably influence drug efficacy and patient responses [171,172,173].

As summarized in Table 2, a variety of studies have explored the relationship between DNA methylation patterns and treatment responses in psychiatric conditions. Depression has been notably highlighted in this context, where baseline hypomethylation of two CpG sites within the serotonin receptor type 1A (HTR1A) and 1B (HTR1B) genes derived from whole blood was predictive of poor escitalopram response in a sample of Han Chinese patients with MDD after 8 weeks of treatment [174]. Furthermore, research on DNA methylation (DNAm) of the BDNF gene in relation to antidepressant treatment response offers varied yet crucial insights. Hypomethylation of BDNF promoter regions has been linked to less favorable responses to a range of antidepressants, including fluoxetine, venlafaxine, and escitalopram, underscoring its potential influence on treatment efficacy [174,175]. On the other hand, studies have shown that higher methylation levels at specific BDNF promoter CpGs correlate with better outcomes, particularly in patients with severe depression treated with escitalopram or venlafaxine, suggesting the modulation of methylation could be a targeted approach for enhancing treatment responses [176,177]. The majority of studies focusing on the genetic basis of antidepressant response have concentrated on genes within the serotonin pathway, specifically the serotonin transporter (SLC6A4) and serotonin receptors (HTR1A/1B). The action of many antidepressants involves the inhibition of SLC6A4, which is crucial for mediating the reuptake of serotonin, while HTR1A and HTR1B receptors are integral in amplifying the therapeutic effects of these drugs [178,179,180]. Recent investigations have expanded the analysis into the interaction of DNAm of HTR1A and HTR1B with environmental factors such as childhood adversity in predicting response to antidepressants. Xu et al. examined DNAm in 181 CpG sites of HTR1A and HTR1B, stratifying their analysis into SSRI and non-SSRI groups. This study revealed that hypomethylation of a particular HTR1A CpG site was associated with an improved response in patients treated with non-SSRI antidepressants (SNRIs and NaSSAs), and those with a specific HTR1B rs6298 SNP and high methylation levels showed better SSRI treatment efficacy. Additionally, an interaction between hypermethylation of HTR1A and high levels of childhood adversity correlated significantly with poor treatment outcomes across both medication groups [181]. Despite extensive research, findings from studies on SLC6A4 methylation and antidepressant treatments, such as SSRIs, TCAs, SNRIs, including escitalopram and venlafaxine, have varied. Initial research in Korean patients suggested minimal hypermethylation at a single CpG site linked with non-response [182], whereas subsequent studies in Japanese and European cohorts reported mixed outcomes, correlating SLC6A4 methylation patterns variably with treatment success [183,184,185,186]. In a subset of five MDD and four panic disorder patients, two CpG sites (14 and 15 in Region A) were significantly hypermethylated following three-month treatment with SSRIs (citalopram, fluoxetine, or sertraline) compared to pre-treatment, although the authors reported that the methodology did not permit reliability of results [187]. Depression was associated with decreased IL6 methylation compared to controls, while antidepressant usage, particularly SSRI-class drugs, was associated with an increase in IL6. [188]. Another study conversely observed decreased IL-6 levels after antidepressant treatment [189]. At the genome scale, a study from Japan investigated the potential of predicting the treatment response to paroxetine based on DNA methylation patterns in the peripheral blood of 68 MDD patients. In their analysis, the authors contrasted the 10 best responders and the 10 worst responders to paroxetine treatment and identified the hypermethylation of the genes liprin-alpha-4 (PPFIA4) and heparan sulfate-glucosamine 3-sulfotransferase 1 (HS3ST1) at baseline in patients with the worst treatment outcomes in relation to the best treatment responders [190]. The role of non-coding RNAs (ncRNAs), particularly microRNAs and long non-coding RNAs, is becoming increasingly recognized within psychiatric pharmacoepigenomics. These ncRNAs modulate transcriptional and translational processes and present novel pathways through which drugs can exert pharmacological effects, independent of direct changes to the DNA sequence [191,192]. More ongoing research efforts aim to identify how these epigenetic mechanisms, influenced or modified by pharmacological treatments, may serve as predictive biomarkers for treatment responses or targets for novel therapeutic interventions. Notably, the use of HDAC and DNMT inhibitors has shown promise in enhancing the efficacy of antidepressants in both preclinical models and clinical settings by modifying epigenetic states [193,194,195,196].

## 7. Pharmacoproteomics in Psychiatry

Pharmacoproteomics is an emerging field within the larger context of biomedical sciences focused on identifying changes in the proteomic landscape as a response to pharmacological treatment. This discipline is particularly relevant in the field of psychiatry, where proteomic profiling has concentrated on elucidating the alterations in specific proteins crucial for cellular communication, signaling, inflammation, and the maintenance and growth of cells following treatment with psychotropic drugs. These insights guide the development of new therapeutic targets, refine drug efficacy assessments, and aid in crafting personalized treatment plans based on protein profiles, thus advancing precision medicine.

Significant advancement has been made in pinpointing proteins that can forecast therapeutic outcomes in psychiatric conditions [198]. For instance, research documented by Schwarz et al. in 2012 identified several proteins, including interleukin-16, fatty acid-binding protein, ferritin, C-reactive protein, myoglobin, prolactin, and complement factor H, that are indicative of symptomatic improvements in schizophrenia patients undergoing antipsychotic treatment [199]. Additionally, other proteins, such as matrix metalloproteinase 2 and insulin, have been associated with enhancements in negative symptoms within this patient population. In the context of depressive disorders, findings from the GENDEP (genome-based therapeutic drugs for depression) study reveal that the levels of C-reactive protein can predict differential responses to pharmacotherapies such as nortriptyline and escitalopram, which have distinct pharmacological profiles [200]. These findings allow clinicians to customize treatments more accurately, potentially minimizing adverse effects and enhancing outcomes.

However, despite these insights, the field has yet to establish proteomic markers validated sufficiently to guide clinical decision-making regarding the selection of psychotropic medications. The GENDEP project consists of a comprehensive series of multidisciplinary studies aimed at discovering predictors of therapeutic response to antidepressants, employing rigorous methodologies across genetics, transcriptomics, and proteomics [201]. For example, a study analyzing antidepressant response in two mouse strains has shed light on molecular mechanisms and potential biomarkers for therapeutic response using advanced quantitative proteomic techniques [202].

Despite its potential, pharmacoproteomics faces hurdles such as the evolving nature of proteomic technologies, the lack of standardized protocols, and the complex proteome of human plasma. Future efforts should focus on rigorous validation across various patient populations and drug classes and establishing interdisciplinary frameworks to standardize proteomic methodologies. By overcoming these challenges, pharmacoproteomics could significantly enhance therapeutic outcomes and minimize adverse effects, thereby refining psychiatric treatment paradigms through precision medicine.

## 8. Harnessing AI and Machine Learning to Enhance Precision Psychiatry Through Multiomics Integration

The burgeoning field of multiomics presents significant complexities due to its expansive and data-rich nature, posing substantial challenges in data integration and interpretation. To manage this abundance of biological data, machine learning and AI have emerged as pivotal tools. These technologies foster novel approaches to effectively integrate disparate omics layers. Advanced computational techniques enable the extraction of meaningful biological insights and facilitate the prediction of disease phenotypes and treatment outcomes, which will enhance precision psychiatry [203,204,205,206,207,208]. This approach leverages detailed molecular and clinical information to craft personalized treatment regimens and improve diagnostic accuracy beyond traditional methods.

### 8.1. Tailoring Treatments in Psychiatry Using Integrated Multiomics Data

In psychiatry, the integration of multiomics data holds potential for tailoring treatments effectively across a spectrum of disorders, such as major depressive disorder (MDD) and bipolar disorder [209,210]. For instance, a study developed predictive models using genomics and plasma metabolomics to forecast outcomes of combination pharmacotherapies in treating MDD. They employed penalized linear regression and XGBoost algorithms to construct two distinct models: one utilizing only metabolomics data, and another integrating both metabolomics and genomics. Their findings indicated that multiomics models achieved superior area under the curve (AUC) measures compared to models using only metabolomics data, underscoring the enhanced accuracy of multiomics in predicting treatment responses [210]. Further supporting this, meta-reviews consolidated findings from various studies, demonstrating how combined multiomics and clinical data can effectively identify predictors of treatment response in unipolar depression [211,212]. Additionally, pharmacogenomic reviews have advocated for including machine learning techniques early in clinical trial planning for psychiatric disorders. These reviews emphasize the significant role of genomic data in elucidating the impact of psychotropic drugs, highlighting studies using machine learning to study genomic influences on the efficacy of treatments for MDD and bipolar disorder using lithium [213,214].

Furthermore, the application of machine learning in psychiatry extends beyond mood disorders. Research demonstrated the use of gradient boosting algorithms to combine exome sequencing data with clinical scores, achieving an AUC of 0.75 in predicting treatment responses in treatment-resistant depression, illustrating the robust potential of integrating genetic data with clinical assessments [215]. Similarly, research on treatment-resistant schizophrenia shows parallel efforts across different psychiatric conditions where predictive models are enriched with omics data [216]. For example, a key study investigated modeling responses to combination antidepressant therapies, examining data from MDD outpatients treated with citalopram or escitalopram in the Mayo Clinic PGRN-AMPS and the CO-MED studies. It focused on enhancing prediction accuracy by integrating metabolomics with validated single-nucleotide polymorphisms (SNPs) compared to using only metabolomics. When trained and tested on both patient cohorts, the models achieved accuracies of 76.6% (AUC: 0.85) without SNPs and 77.5% (AUC: 0.86) with SNPs, demonstrating robust cross-trial prediction replication [210]. In a notable study, Lin et al. [217] utilized a sophisticated deep learning framework that combines genetic data such as SNPs, demographic factors like age and sex, and clinical insights including depression severity scores and suicide attempt statuses in a genome-wide association study (GWAS). Employing multi-layer feedforward neural networks (MFNNs) equipped with multiple hidden layers, they achieved promising predictive accuracies, demonstrating the capability of deep learning frameworks to capture the complex interplay between diverse biomarkers and treatment outcomes, thus offering enhanced predictive precision essential for clinical applications. Additionally, various studies have explored the use of conventional AI and machine learning techniques, such as random forest and decision tree algorithms, to improve the predictability of antidepressant responses. These methods analyze combined datasets consisting of genetic, demographic, and clinical data, generating new insights into patient-specific treatment outcomes and enhancing the potential of personalized psychiatric treatment protocols [205,206,207,208,218].

Table 3 provides a comprehensive summary of pivotal studies involving AI and machine learning in psychiatry, specifically focusing on predictive modeling for treatment responses in psychiatric disorders.

### 8.2. Challenges in AI and Machine Learning Deployment for Personalized Psychiatry

The deployment of AI and machine learning presents striking predictive possibilities that are tempered by compelling limitations. One of the most significant challenges is the “black box” nature of these models, where the lack of transparency in decision-making processes can hinder trust and understanding among clinicians and patients, thereby affecting their ethical integration into clinical practice. Additionally, these models are constrained by data heterogeneity and potential biases; models trained on non-representative datasets may yield skewed predictions that exacerbate health disparities. The issue of generalizability also arises, as models developed in specific clinical or demographic environments often fail to perform accurately across different populations due to genetic, environmental, and disease prevalence variations. Interoperability issues and ethical concerns such as data privacy, autonomy, and informed consent present further barriers to integration within existing healthcare systems. Ethical deliberations in the deployment of AI and machine learning in healthcare encompass examining biases in training datasets and algorithmic decisions, ensuring transparency in AI methodologies, clarifying accountability for AI-driven outcomes, and safeguarding patient privacy. These considerations are crucial, as they address fundamental ethical principles and help foster a climate of trust and reliability in AI and machine learning applications. Critical questions about how biases within these systems are identified and mitigated, the extent of transparency required to maintain patient and clinician trust, how accountability is apportioned in cases of AI error, and the mechanisms protecting patient data must be rigorously examined and addressed by a multidisciplinary team. Furthermore, ethical concerns extend to issues of equity and justice, particularly examining who benefits from these advancements and who may be inadvertently harmed. The emotional and psychological impacts of AI and machine learning decisions on patients also require careful attention, especially in fields like psychiatry, where the implications of errors are profound [219,220,221]. These challenges underscore the necessity for a multidisciplinary approach involving data scientists, clinicians, ethicists, and policymakers to forge AI and ML tools that are transparent, equitable, adaptable, and aligned with both ethical standards and societal values, thereby ensuring their successful implementation in personalized psychiatry.

**Table 3:** Representative studies on AI and machine learning applications for predictive modeling in psychiatric treatment responses.

Reference	Sample Description	Outcome Measure	Model(s) Used	Primary Performance Metric	Data Used
Lin et al. [217]	MDD patients Antidepressants	Treatment response (8 weeks)	Deep learning architecture	AUC: 0.82 for treatment response, 0.81 for remission	-
Joyce et al. [210]	MDD patients (PGRN-AMPS) SSRI	Treatment response (8 weeks)	Linear penalized regression, XGBoost	AUC (multiomics > metabolomics)	Multiomics, metabolomics
Chekroud et al. [218]	MDD patients; various antidepressants	Remission (12 weeks)	Tree-based ensemble	Accuracy: 0.59	Top 25 predictors
Chang et al. [204]	MDD patients; antidepressants	Treatment response (8 weeks)	Linear regression (ARPNet)	Accuracy: 0.84	Neuroimaging biomarkers, genetic variants, DNA methylation, demographics information
Athreya et al. [203]	MDD patients; SSRI	Treatment response (8 weeks)	Random forest	AUC: >0.7, accuracy: >0.69	SNP datasets
Eugene et al. [222]	Bipolar patients Lithium	Treatment response	Decision tree, random forest	AUC:0.92 (male; 2 genes) AUC: 1 (female; 3 genes)	Gene expression data
Cepeda et al. [223]	Patients with and without proxy for treatment-resistant depression (TDR); antidepressants and antipsychotics	Treatment resistance (12 months)	Decision tree	AUC: 0.81	Health claims databases (CCAE, MDCR, Optum)
Kautzky et al. [224]	MDD patients; antidepressants	Treatment resistance	Random forest	Accuracy: 0.73 (TRD); accuracy: 0.85 (remission)	Not specified
Pradier et al. [225]	Bipolar patients; antidepressants	Conversion to bipolar diagnosis within 3 months	LASSO logistic regression, random forest	AUC: 0.80	-

## 9. Challenges and Future Directions in Personalized Psychiatry

Personalized psychiatry stands at the cusp of a transformative era, significantly propelled by the integration of multiomics approaches. These methods are pioneering enhanced understanding and fostering the development of more effective treatment modalities for psychiatric conditions. However, translating such advanced methodologies into clinical paradigms presents formidable challenges. The inherent complexity and considerable costs associated with multiomics technologies [218] necessitate the implementation of sophisticated, adaptable analytical frameworks. Traditional genomic analyses often fall short in capturing dynamic interactions between biological and environmental factors, further emphasizing the need for advancements in this domain.

### 9.1. Global Efforts and Initiatives

Amid these challenges, a broad spectrum of coordinated, multi-level efforts underpins the progression of personalized psychiatry. Key global consortiums such as the Psychiatric Genomics Consortium (PGC) [226], the European College of Neuropsychopharmacology (ECNP) [227], and the UK Biobank [228] play pivotal roles in amassing extensive biological and phenotypic datasets from various populations. These data are crucial for delineating the intricate interplay of genetic, environmental, and lifestyle factors. Concurrently, established clinical guidelines set forth by authoritative entities such as the Canadian Pharmacogenomics Network for Drug Safety (CPNDS) [229] and the French National Network of Pharmacogenetics (RNPGx) [230], endorsed by organizations like the American Society of Health-System Pharmacists (ASHP) [231], guide the safe and effective integration of genomic insights into clinical frameworks. Moreover, implementation networks such as IGNITE actively address logistical and operational hurdles in genomic medicine, utilizing tools like the Consolidated Framework for Implementation Research (CFIR) to facilitate practical applications [232,233]. On the frontier of innovation, projects like the PRIME Care program [234] and the PREPARE study [235] within the U-PGx initiative lead the way in integrating preemptive genotyping and pharmacogenomics into clinical trials and healthcare practice. These initiatives and efforts are reinforced by advancements related to the progress of AI/ML in precision medicine, driven by collaborative endeavors across the modeling community, regulatory bodies, and scientific institutions. The Innovation and Quality (IQ) Consortium’s 2022 workshop exemplifies this synergy, promoting AI/ML adoption and advocating for the integration of domain-specific knowledge to enhance model applicability in pharmaceutical research [236]. The prevailing approach favors synergistic integration where AI/ML complements existing pharmacokinetic–pharmacodynamic (PK-PD) models rather than replacing them, thereby enhancing predictive accuracy. This method aggregates AI’s robust data processing capabilities with the biological insights of traditional models, exemplified by hybrid ML-PK/PD/toxicodynamic approaches that optimize therapeutic strategies using complex biomedical data. Regulatory engagement, illustrated by the collaborative workshop with the FDA and the University of Maryland, further highlights AI/ML’s significant role in refining patient-specific treatments [237]. The increasing number of AI/ML-related regulatory submissions to the FDA underscores the growing trust in these technologies to improve clinical trial designs, patient stratification, and treatment adherence [238]. Additionally, the advancement of emerging disciplines such as connectomics, a nascent field crucial for mapping neural connections and understanding how various psychiatric treatments interact with brain systems [239], and the implementation of pharmacogenomic (PGx) scoring systems [240] are integral to ongoing efforts aimed at refining therapeutic approaches for complex psychiatric disorders. These scientific advancements are crucial in aligning with and pushing the frontiers of personalized medicine to develop more targeted and effective treatment protocols for a range of psychiatric conditions.

### 9.2. Considerations and Perspectives

These multifaceted initiatives exemplify a robust, concerted endeavor to overcome existing barriers such as high costs, technological complexity, and disparate healthcare infrastructures.

Financially, the utilization of omics technologies necessitates significant investment in both cutting-edge equipment and specialized personnel training, making it prohibitive for many institutions, especially those in resource-limited settings. Technologically, the integration and standardization of sophisticated genomic data systems require advanced IT infrastructure and data management skills that are not uniformly available across different healthcare providers. Regional disparities in healthcare infrastructure further accentuate these challenges, with differences in healthcare quality, technological access, and professional training potentially creating significant gaps in the effectiveness of these strategy applications. Such disparities necessitate tailored implementation strategies that consider the specific capacities and needs of local contexts. Ethical considerations also come to the forefront, particularly concerning the security of patient data, potential psychosocial effects, and the risk of discrimination. To ensure the responsible implementation of clinical applications, it is vital that decisions be based on solid evidence weighing potential risks against benefits. This involves strict data governance, comprehensive patient education, and the use of explainable AI and machine learning models. Additionally, policy frameworks must promote equitable access, particularly for vulnerable groups, to mitigate the risk of widening health disparities. Integrating insights from all stakeholders and maintaining transparency throughout all phases of implementation are essential, complemented by continuous validation and cost-effectiveness analyses to ethically advance precision psychiatry. The future success of these endeavors hinges on sustained investments in technological innovation, interdisciplinary research, and global collaboration. Knowledge exchange, facilitated through symposia, workshops, and publications, along with equipping clinicians with necessary tools for targeted interventions, holds the promise of reshaping psychiatric practices. Ultimately, the aim is to meet the unique treatment needs of each patient more effectively and precisely, thereby emphasizing the critical importance of continued validation, research, and implementation of these sophisticated strategies.

## 10. Conclusions

**Figure 1:**
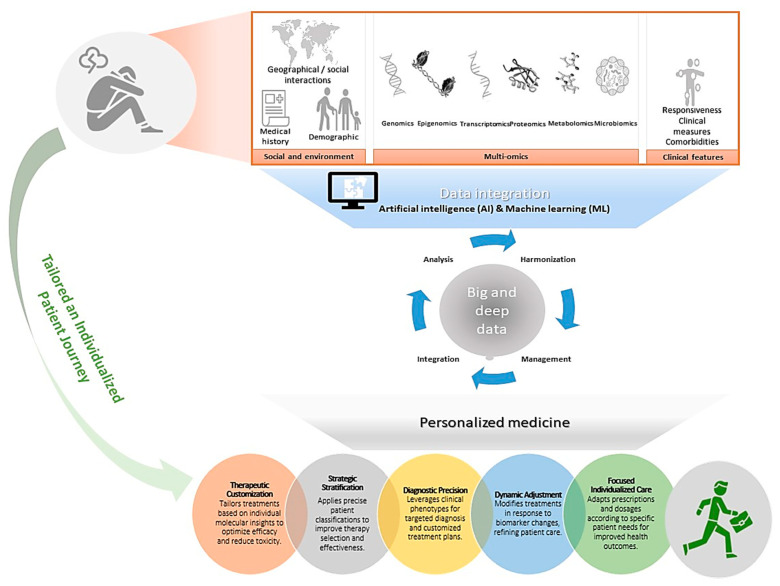
Navigating the path to precision psychiatry. This figure outlines a novel approach in personalized psychiatry, predicated on the integration of diverse datasets and encompassing pharmaco-multiomics. Utilizing advanced AI and ML technologies, these integrated data are leveraged to yield precise insights that enable the formulation of tailored therapeutic interventions and dynamic management strategies. This methodology distinctly addresses the intricate needs of psychiatric care and emphasizes patient-centered care.

In this review, we have delved into the multifaceted realm of pharmaco-omics and examined its pivotal contributions to demystifying the complexities inherent in psychiatric disorders. We emphasize the transformative potential of integrating diverse omics fields, including genomics, proteomics, transcriptomics, the microbiome, and metabolomics, into a unified analytical framework. This holistic approach advocates for a personalized treatment strategy meticulously tailored to each patient’s unique biological profile, moving past the conventional trial-and-error methods prevalent in psychiatric practice. Looking to the future, the ability of pharmaco-multiomics to revolutionize psychiatric treatment relies on overcoming several critical challenges. These include the integration of complex multiomics data to yield clinically actionable insights, bridging technological gaps, and upholding ethical standards in data management and privacy (Figure 1). To fully realize and harness the capabilities of a personalized pharmaco-multiomics approach in psychiatry, key strategic measures are crucial:

- Interdisciplinary Collaborations: Establish robust collaborations among researchers, clinicians, bioinformaticians, and ethicists. Such interdisciplinary partnerships are essential for developing treatment modalities that are predictive, preventive, and deeply personalized.
- Investment in Technological Infrastructure: Commit substantial investment into developing technological infrastructures and research ecosystems. This is particularly critical in regions like the Middle East, where numerous challenges obstruct the adoption of advanced multiomics strategies.
- Enhanced International Cooperation: Strengthen international cooperation to facilitate efficient data sharing and the pooling of global expertise. Such collaboration is vital for leveraging diverse knowledge and resources to advance psychiatric treatment modalities.
- Integration of AI and Machine Learning: Deepen the integration of AI and machine learning technologies within pharmaco-multiomics. These technologies are key to managing large, complex datasets and enabling sophisticated predictive modeling that can inform personalized treatment plans.
- Overcoming Methodological Barriers: Address critical barriers such as data diversity, sample size, and methodology standardization. It is imperative to resolve these issues to ensure that research findings are replicable and interpretable across different settings.
- Translation of Multiomics Data: With a robust technological and collaborative framework in place, focus on the effective integration of multiomics approaches to convert complex biological data into tailored therapeutic strategies. This will facilitate the creation of customized treatment plans that meet individual patient needs.

By overcoming existing challenges and implementing key strategic measures, we can fully unlock the potential of pharmaco-multiomics. This heralds a new era in personalized psychiatry that respects the complex biological individuality of each patient, signifying substantial scientific progression and shifting toward a more compassionate, individualized, and effective approach to mental health care.

## Figures and Tables

**Table 1:** Key genes and guidelines for psychotropic medications (PharmGKB).

Class	Drug	Informative PGx (PharmGKB Level 2, 3, and 4)	Actionable PGx (PharmGKB Level 1)	Variant	Recommendation	CPIC/DPWG
Antidepressants	Citalopram	*ABCB1* *BDNF* *CACNA1C* *COL26A1* *CREB1, METTL21A* *CRHR2* *CYP2D6* *DTNBP1* *ERICH3* *FKBP5* *GLDC* *GRIA3* *GRK5* *GSK3B* *HTR1B* *HTR2A* *NEDD4L* *PAPLN* *REEP5* *RFK* *RORA* *SERPINE1* *SRP19* *TPH2* *BDNF* *CYP2D19* *CYP2D6* *HTR2A* *SLC6A4* *TPH1* *TSPAN5*	*CYP2C19*	CYP2C19*1; CYP2C19*2; CYP2C19*3; CYP2C19*17 CYP2C19*4	UM, LP, and PM: consideration of a clinically appropriate antidepressant not predominantly metabolized by CYP2C19 for CYP2C19 In case citalopram or escitalopram are clinically appropriate, dose alterations are recommended.	CPIC
Antidepressants	Escitalopram	*BDNF* *BMP5* *CYP1A2* *CYP2D6* *ERICH3* *FKBP5* *GLDC* *GRK5* *HTR1B* *HTR2A* *HTR2C* *HTR7* *IL11* *RFK* *SCL6A4* *TSPAN5*	*CYP2C19*	CYP2C19*1; CYP2C19*2; CYP2C19*3; CYP2C19*17	UM, LP, and PM: consideration of a clinically appropriate antidepressant not predominantly metabolized by CYP2C19 for CYP2C19 In case citalopram or escitalopram are clinically appropriate, dose alterations are recommended.	CPIC
Antidepressants	Fluvoxamine	*ABCB1* *COMT* *FGF2* *HTR1A* *MDGA2* *SLC6A4* *HTR2A*	*CYP2D6*	CYP2D6*1; CYP2D6*4; CYP2D6*5; CYP2D6*6; CYP2D6*10; CYP2D6*14	PM: 25–50% reduction in recommended starting dose and slower titration schedule or use of an alternative drug not metabolized by CYP2D6	CPIC
Antidepressants	Paroxetine	*ABCB1* *BDNF* *COMT* *CYP1A2* *DRD3* *FKBP5* *GDNF* *HTR1A* *HTR1B* *HTR2A* *HTR7, RPP30* *MDGA2* *REEP5* *SLC6A4* *SRP19* *HTR2A* *HTR3B* *TPH1*	*CYP2D6*	CYP2D6*1; CYP2D6*1xN; CYP2D6*2; CYP2D6*2xN; CYP2D6*3; CYP2D6*4; CYP2D6*5; CYP2D6*9; CYP2D6*10; CYP2D6*14; CYP2D6*41	UM: alternative drug not predominantly metabolized by CYP2D6. PM: 50% reduction in recommended starting dose, slower titration schedule, and a 50% lower maintenance dose	CPIC
Antidepressants	Sertraline	*ABCB1* *ACE* *CYP2D6* *GNB3, P3H3* *HTR1A* *REEP5* *SRP19* *HTR2A* *SLC6A4*	*CYP2C19, CYP2B6*	CYP2B6*1; CYP2B6*4; CYP2B6*6; CYP2B6*9 CYP2C19*1; CYP2C19*2; CYP2C19*3; CYP2C19*17	CYP2C19 PM: 50% reduction in recommended starting dose and titrate to response or selection of an alternative drug not predominantly metabolized by CYP2C19	CPIC
Antidepressants	Imipramine	*-*	*CYP2C19, CYP2D6*	CYP2C19*1; CYP2C19*2; CYP2C19*3; CYP2C19*17 CYP2D6*1; CYP2D6*3; CYP2D6*4; CYP2D6*5; CYP2D6*6; CYP2D6*1xN; CYP2D6*2xN	CYP2D6 IM: a 25% dose reduction should be considered.	CPIC
Antidepressants	Clomipramine	*ABCB1* *FKBP5* *HTR1B* *SLC6A4*	*CYP2C19, CYP2D6*	CYP2C19*1; CYP2C19*2; CYP2C19*3; CYP2C19*17 CYP2D6*1; CYP2D6*1xN; CYP2D6*2; CYP2D6*3; CYP2D6*4; CYP2D6*5; CYP2D6*6; CYP2D6*10; CYP2D6*41	IM: 25% dose reduction	CPIC
Antidepressants	Nortriptyline	*-*	*CYP2D6*	*-*	IM: 25% dose reduction UM/PM: alternative drug should be considered. If nortriptyline is warranted, consider a 50% dose reduction in CYP2D6-poor metabolizers.	CPIC
Antidepressants	Amitriptyline	*ABCB1* *CYP2D6* (CYP2D6*1, CYP2D6*87, CYP2D6*88, CYP2D6*89, CYP2D6*90,CYP2D6*91,CYP2D6*93, CYP2D6*94, CYP2D6*95, CYP2D6*97, CYP2D6*98)	*CYP2C19, CYP2D6*	CYP2C19*1; CYP2C19*2; CYP2C19*3; CYP2C19*17 CYP2D6*1; CYP2D6*1xN; CYP2D6*2; CYP2D6*3; CYP2D6*4; CYP2D6*5; CYP2D6*6; CYP2D6*10; CYP2D6*41	CYP2D6 UM/PM or CYP2C19 UM/RM/PM: alternative drug PM: If amitriptyline is warranted, consider a 50% dose reduction. CYP2D6 IM: a 25% dose reduction should be considered.	CPIC
Antidepressants	Venlafaxine	*ABCB1* *COMT* CYP2C19 (CYP2C19*2) CYP2D6 (CYP2D6*1, CYP2D6*87, CYP2D6*88, CYP2D6*89, CYP2D6*90, CYP2D6*91, CYP2D6*93, CYP2D6*94, CYP2D6*95, CYP2D6*97, CYP2D6*98) *FKBP5* *GABRA6* *GABRP* *GABRQ* *GRIA3* *HTR2A* *SLC6A2* *TPH2* CYP2C19 (CYP2C19*1; CYP2C19*2) *HTR2A* *SLC6A4*	*CYP2D6*	CYP2D6*1; CYP2D6*3; CYP2D6*4; CYP2D6*6; CYP2D6*81; CYP2D6*5; CYP2D6*10	PM: alternative antidepressant not predominantly metabolized by CYP2D6	CPIC
Antipsychotics	Aripiprazole	*ABCB1* *ANKK1* *DRD2* *FAAH* *MC4R* *RABEP1* *SH2B1* *TAAR6*	*CYP2D6*	CYP2D6*1; CYP2D6*4; CYP2D6*5; CYP2D6*6; CYP2D6*10; CYP2D6*41	PM: reduce maximum dose	DPWG
Antipsychotics	Risperidone	*ABCB1* *ADRB2* *AKT1* *ANKK, DRD2* *CCL2* *CNR1* *COMT* *CYP1B1* CYP2D6 (CYP2D6*1, CYP2D6*87, CYP2D6*88, CYP2D6*89, CYP2D6*90,CYP2D6*91, CYP2D6*93, CYP2D6*94, CYP2D6*95, CYP2D6*97, CYP2D6*98) *CYP2E1* *DRD2* *DRD3* *EIF2AK4* *EPM2A* *FAAH* *GRID2* *GRIN2B* *GRM3* *GRM7* *HRH3* *HRH4* *HTR1A* *HTR2A* *HTR2C* *HTT, MSANTD1* *LEP* *MC4R* *NR1I2* *PPA2* *RABEP1* *RGS4* *SH2B1* *SLC6A4* *TJP1* *TNFRSF11A* *UGT1A1,* UGT1A10,UGT1A3, UGT1A4, UGT1A5, UGT1A6, UGT1A7, UGT1A8, UGT1A9 ABCB1 *ANKK1, DRD2* *COMT* *DRD2*	*CYP2D6*	CYP2D6*1; CYP2D6*1xN, CYP2D6*3; CYP2D6*4; CYP2D6*5; CYP2D6*6; CYP2D6*10; CYP2D6*14	PM: decrease dose UM: alternative drug or titration of the dose according to the maximum dose of the active metabolite	DPWG
Antipsychotics	Zuclopenthixol	*-*	*CYP2D6*	CYP2D6*1, CYP2D6*3, CYP2D6*4	PM/IM: dose reduction UM: increase dose (not exceeding 1.5x normal dose) if needed	DPWG
ADHD	Atomoxetine	CYP2D6 (CYP2D6*1, CYP2D6*87, CYP2D6*88, CYP2D6*89, CYP2D6*90, CYP2D6*91, CYP2D6*93, CYP2D6*94, CYP2D6*95, CYP2D6*97, CYP2D6*98, CYP2D6*4, CYP2D6*5) *SLC6A2*	*CYP2D6*	CYP2D6*1; CYP2D6*3; CYP2D6*4; CYP2D6*4xN; CYP2D6*5; CYP2D6*6; CYP2D6*10; CYP2D6*17; CYP2D6*92; CYP2D6*96	UM: be alert to reduced efficacy or select alternative drug as precaution PM: be alert to side effects	DPWG
Anticonvulsants	Carbamazepine	***SCN1A*** *ABCB1* *ABCC2* *BAG6, PRRC2A* *CYP1A1* *CYP1A2* *CYP3A4* *CYP3A5* *EPHX1* *GABRA1* HLA-A (HLA-A*24:02; HLA-A*11:01, HLA-A*02:01) HLA-B (HLA-B*46:01, HLA-B*15:18, HLA-B*15:21, HLA-B*51:01, HLA-B*59:01, HLA-b*58:01, HLA-B*40:01) *HLA-C* *HLA-DRB1* *HSPA1A, HSPA1L* *LTA, TNF* *NR1I2* *SCN2A* *UGT2B7* *HNF4A* *SCN1A* *SCN2A*	*HLA-A, HLA-B*	HLA-A*31:01 HLA-B*15:02; HLA-B*15:11	Alternative drug for carbamazepine-naive patients carrying at least one copy of either HLA-B*15:02 or HLA-A*31:01	CPIC
Anticonvulsants	Oxcarbazepine	*ABCB1* *HLA-A* HLA-B (HLA-B*27:09, HLA-B*13:02, HLA-B*38:02, HLA-B*48:04, HLA-B*15:19, HLA-B*15:27, HLA-B*40:02, HLA-B*15:18, HLA-B*40:01, HLA-B*15:02) *HLA-DRB1* *SCN2A* *UGT1A10, UGT1A7, UGT1A8, UGT1A9* *UGT2B7* *ABCC2* *SCN1A*	*HLA-B*	B*15:02	Alternative drug for oxcarbazepine-naive patients carrying at least one copy of HLA-B*15:02	CPIC

**PharmGKB:** Pharmacogenomics Knowledgebase; **PGx:** pharmacogenes; **CYP:** cytochrome P450; **UM:** ultra-rapid metabolizer; **IM: intermediate metabolizer**; **LP:** likely poor metabolizer; **PM:** poor metabolizer; **CPIC:** Clinical Pharmacogenetics Implementation Consortium; **DPWG:** Dutch Pharmacogenetic Working Group.

**Table 2:** Overview of key studies investigating DNA methylation patterns associated with response to psychiatric treatments.

Reference	Participant	Treatment(s)	Duration (Weeks)	Outcome Measures	Genetic Marks Assessed	Sample Type	Key Findings
Hsieh et al. [197]	MDD patients (Taiwan)	SSRIs and benzodiazepines or hypnotics (no mood stabilizers or antipsychotic drugs)	4	HAM-D17	14 CpG sites in *BDNF* exon IX	Peripheral blood	Higher methylation level at CpG 24 and CpG 324 of *BDNF* exon IX associated with improved response
Schiele et al. [186]	MDD patients (Caucasian)	Antidepressants	6	HAM-D21	9 CpG sites in SLC6A4 promoter region	Blood cells	Higher methylation of CpG sites correlated with treatment response
Xu, Chen, et al. [181]	MDD patients (Chinese, Han)	Antidepressants	2	HAM-D17 LES CTQ	181 CpG sites in *HTR1A* and *HTR1B*	Peripheral blood	Methylation of HTR1A-2-143 and HTR1B-3-61 associated with response; interaction with rs6298 genotype
Wang, Zhang, et al. [174]	MDD patients (Chinese, Han)	Escitalopram	8	HAMD-17 LES CTQ	90 CpG sites in *BDNF*	Peripheral blood	Methylation at four *BDNF* amplicons associated with response (higher DNA methylation associated with better treatment response)
Takeuchi et al. [190]	MDD patients (Japanese)	Paroxetine	6	HAM-D 21 change	Genome-wide DNA methylation	Peripheral blood	Methylation of *PPFIA4* CpG cg00594917 and *HS3ST1* CpG cg07260927 associated with response

## Abbreviations

The following abbreviations are used in this manuscript:

CBZ	Carbamazepine
CPIC	Clinical Pharmacogenetics Implementation Consortium
DNAm	DNA methylation
DPWG	Dutch Pharmacogenetics Working Group
FDA	U.S. Food and Drug Administration
HLA	Human leukocyte antigen
MDD	Major depressive disorder
ncRNA	Non-coding RNA
PGx	Pharmacogenomics
PharmGKB	Pharmacogenomics Knowledgebase
PMs	Poor metabolizers
SNP	Single-nucleotide polymorphism
SSRIs	Selective serotonin reuptake inhibitors
UMs	Ultra-rapid metabolizers
VPA	Valproic acid
